# Web Survey of Turkish Pharmacy Students Comparing First and Fifth Years’ Antidepressant Awareness and Stigmatizing Attitudes Regarding Depression and Anxiety

**DOI:** 10.3390/pharmacy12020045

**Published:** 2024-03-01

**Authors:** Nadir Yalçın, Gökçe Gül Özkan, Karel Allegaert, Sertaç Ak, Kutay Demirkan

**Affiliations:** 1 Department of Clinical Pharmacy, Faculty of Pharmacy, Hacettepe University, 06230 Ankara, Türkiye; kutay@hacettepe.edu.tr; 2 Faculty of Pharmacy, Hacettepe University, 06230 Ankara, Türkiye; gokcegul.38@hotmail.com; 3 Department of Pharmaceutical and Pharmacological Sciences, 3000 Leuven, Belgium; karel.allegaert@kuleuven.be; 4 Department of Development and Regeneration, 3000 Leuven, Belgium; 5 Department of Hospital Pharmacy, Erasmus Medical Center, 3015GD Rotterdam, The Netherlands; 6 Department of Psychiatry, Faculty of Medicine, Hacettepe University, 06230 Ankara, Türkiye; sertac@hacettepe.edu.tr

**Keywords:** antidepressants, pharmacy education, pharmacy students, stigma, depression, anxiety, peer use, stigmatizing attitudes

## Abstract

The prevalence of depression and anxiety has increased day by day. Prejudice, self-stigma, and public stigma, on the other hand, continue to prevent patients from seeking adequate treatment, particularly in traditional communities. In this web-based, cross-sectional study, both the presence of depression and anxiety, and the knowledge, attitude, and awareness of first- and fifth (final)-year pharmacy students were examined via an online survey. The aim was to demonstrate the potential impact of public information and five years of pharmacy school on knowledge, attitude, and awareness. Our study population consisted of first- and fifth-year pharmacy students enrolled in one faculty of pharmacy during the spring semester of 2022–2023. The Beck Depression Inventory and Beck Anxiety Scale were utilized to measure the presence of depression and anxiety, while the Depression and Antidepressant Awareness and Knowledge Scale (DAKAS) was applied to assess their knowledge, attitude, and awareness. Fifth-year participants (n = 101) exhibited noticeably fewer stigmatizing attitudes than first-year participants (n = 104) (*p* < 0.05). There was no statistically significant difference between the mean Beck Depression Inventory and Beck Anxiety scores in first- and fifth-year pharmacy students. Being in the fifth class (OR: 3.690; *p* = 0.025), being of female gender (OR: 4.653; *p* < 0.001), and having a relationship with someone who took a psychotropic (OR: 3.060; *p* = 0.008) were associated with a lower overall stigma score by multiple linear regression analysis. The students’ awareness of antidepressants and familiarity with mental health issues at the end of their pharmacy education were higher and stigmatization behavior was lower than in first-year students. The positive attitudes at the end of their training towards depression will reduce the likelihood of future pharmacists’ patients from being exposed to stigmatization, prevents the formation of an additional stress factor, and likely will improve pharmacy practices.

## 1. Introduction

The prevalence of mental health conditions is increasing [1]. Antidepressant use is growing along with this increase, with depression and anxiety disorders as the most prevalent [2,3]. The consumption of antidepressants in Turkey increased by 3.6 dosages per 1000 inhabitants per day (+7.36 percent) in 2021 compared to the previous year. In total, the consumption amounted to 52.5 dosages per 1000 inhabitants per day in 2021, which was the highest figure recorded over the observed period [4]. Despite this increase, patients continue to face barriers to have access to appropriate care due to persistent prejudice [5,6,7], self-stigma, or public stigma [8,9]. According to a survey of Turkish medical students, 70.4–78.9% of respondents think that social stigmatization has a detrimental effect on adherence to antidepressants [10].

Pharmacy students are in the high-risk category for depression and anxiety [11]. It is known that approximately one in four pharmacy students report clinically significant symptoms of anxiety and depression with a cumulative effect throughout the school years [12,13]. In a systematic review on the prevalence of depressive symptoms, severe symptoms were observed in up to 50% of pharmacy students [12,14]. Furthermore, this group includes health professionals such as future community, clinical, and industrial pharmacists. Obviously, pharmacists’ awareness, attitude, and level of knowledge on mental health conditions and pharmacotherapy have an impact on the mental health of their patients [15,16]. 

In mental health conditions, stigma may have significant impacts. For example, people affected may experience troubling symptoms of the condition itself, may delay help-seeking, and experience labelling, prejudice, and stereotypes from community members, including those working within a healthcare system, and some health professionals [17,18].

Studies examining the knowledge, awareness, and stigma around antidepressants in pharmacists are extremely rare, despite the fact that many studies report on public levels of depression and general attitudes of depression [19]. The opportunity to reduce the potential experience of stigma from future health professionals can benefit both the individual living with a mental health issue and the health professionals. Understanding the challenges young people face with stigma can enable us to create interventions that motivate them to openly address their mental health concerns, facilitating access to the necessary support and treatment [20]. To the best of our knowledge, although there are many studies on the mental health literacy and stigma towards patients in pharmacists, pharmacy students have not been the subject of such a study [21,22,23,24]. This is of specific relevance for pharmacy students, due to its socio-environmental factors and its cultural diversity bridging between Europe and Asia. At the same time, Turkey is key because of the presence of several other ethnic minorities. Based on this, we have the following hypotheses:

*Primary hypothesis:* With the maturation brought by the increase in age, participation in pharmacy and medical courses, and the contribution of clinical pharmacy courses, there are higher levels of depression and anxiety, a greater knowledge and understanding of depression and antidepressants, and therefore decreased stigmatizing views among fifth-year students from pharmacy school as compared to first-year students.

*Secondary hypothesis:* As pharmacy students become more familiar with mental health conditions and treatments, we anticipate that stigmatizing perceptions will change between the beginning and end of training due to the increase in the number of psychiatrist and psychologist consultations of students, their relatives, and friends. These implications may affect the students’ approach to their patients after graduation.

## 2. Materials and Methods 

### 2.1. Participant Characteristics

The study’s participants as the target population were recruited from first- and fifth-year students at Hacettepe University’s Faculty of Pharmacy in Ankara, Turkey, via social platforms such as WhatsApp (23.19.0), Facebook (453.0.0), and Twitter (3.1). With students coming from all over the nation from various areas and with a variety of backgrounds, the pharmacy school at Hacettepe University serves as an excellent cross-section and focal point for Turkish pharmacy students as well as the Turkish people. The fact that Hacettepe University ranked first in Turkey and in the 201–250 rankings in the world in the 2023 QS World University Rankings in the category of pharmacy and pharmacology shows that it has a great socio-cultural role in its popularity and is the most preferred pharmacy school in our country [25,26].

### 2.2. Survey Tool and Measurements

The study design was a web-based, cross-sectional study with sufficient detail to allow others to replicate and build on the published results. The sampling procedure was a non-probability voluntary (self-selection) sampling method. The data collection period spanned 4 months, with an additional 2 months allocated for data analysis. 

The Google^®^ Forms open survey creation tool was used to construct the questionnaire’s online version and sent to potential participants via social platforms (WhatsApp, Facebook, Twitter) of student groups/pages online. There was no randomization of items or questionnaires, and the responses were captured using an automated method. 

At the beginning of the survey, participants were informed about the estimated duration, where and for how long the data would be stored, the identity of the researcher, and the purpose of the study. The initial contact and survey announcements with potential participants were conducted face-to-face during the course by teaching staff. Participants’ identity information (name-surname, student number, etc.) was not questioned throughout the survey. The questionnaire data were stored in the password-protected Google^®^ Forms until the targeted sample size and then converted into SPSS format and stored in the password-protected computer folder of the responsible researcher. The questionnaire was completed in the classroom under the guidance of the responsible researcher by giving sufficient time to complete the questionnaire. There was no incentive to participate, and a single anonymized reminder was sent one week later for students who did not participate in the survey. The study protocol was approved by the Local Ethics Committee (decision no. 2023/01-61). The survey tool took 15 min to complete according to several respondents’ feedback, and contained a sociodemographic information form including the socio-cultural role of the students, as well as questionnaires on depression, anxiety, and the Depression and Antidepressant Knowledge and Awareness Scale (DAKAS) as one page for each of these items. The questionnaire consists of 16 pages in total. The survey design did not contain filters or contingency items and did not account for the hosting of the survey, and the answers progressed the participants further into the survey. In this survey, the selection of one response option was enforced.

“The Checklist for Reporting Results of Internet E-Surveys (CHERRIES)” has been used to improve the reporting of our online survey (Supplementary Material).

The sociodemographic information form, which included questions about one’s self-reported gender and family status, and specific questions about one’s personal and family history of mental health conditions, was developed by the researchers by considering the characteristics of the study population, and then validated by content validity by these researchers. 

The 21-question Beck Depression Inventory (BDI) [27], which measures characteristic attitudes and symptoms of depression with a self-report, was used with an adopted Turkish version [28]. In this scale, each question is scored between 0 (lowest) and 3 (highest) levels. Conventional cut-offs encompass 0–9 to denote a normal range, 10–18 to indicate mild to moderate depression, 19–29 for moderate to severe depression, and 30–63 for severe depression [29]. Also, the 21-question Beck Anxiety Scale (BAS) [30], which measures somatic symptoms of anxiety such as dizziness, nervousness, and the inability to relax with a self-report, was used with an adopted Turkish version [31]. In this scale, each question is scored between 0 (lowest) and 3 (highest) levels. A total score falling within the range of 0–7 is categorized as minimal, 8–15 as mild, 16–25 as moderate, and 26–63 as severe [32].

The DAKAS developed by Nalcakan et al. focuses on the use of antidepressants and some specific perspectives on depression [10]. The scale consists of two parts:

- A stigma component (Section A). In this section, participants were asked to express their opinions on the items using a 5-point Likert scale (strongly agree/agree/neutral/disagree/strongly disagree). The first 14 questions in Section A were added together to determine the overall stigma score. The final two items (items 16 and 17), which featured expressions that are often used but are not a good indicator of stigma and were therefore deleted, are crucial for understanding the study participants’ thoughts. In item 15, the term “numb” was omitted from the overall stigma score due to its potential to be interpreted as either emotional numbness or the anesthesia effect of narcosis in Turkish. This decision aimed to avoid confusion for both the creators and participants [10].
- Section B focuses on general standardized knowledge questions about depression and antidepressants in particular. The statements are simple sentences that can be responded to with “yes”, “unsure”, or “no”. Common information on these themes is provided in this section to assist respondents in their responses.

### 2.3. Sample Size Estimation

Based on a previously published study in medical students [10], it was predicted to reach a total of 218 participants, 109 participants in each of the two classes, with an effect size of 0.36, 95% power, and 5% margin of error during the study period (G*Power Analysis 3.1.9.6).

### 2.4. Statistical Analysis

Continuous variables were described as the mean (standard deviation, SD) or median (range). Categorical variables were described as the frequency and percentage. The normality of continuous variables was tested using the Shapiro–Wilk test. The relationships between categorical variables were evaluated by the Chi-Square test. When parametric test assumptions were met, comparisons between two independent groups were made with the independent sample T-test. Otherwise, the Mann–Whitney U test was used. The comparison of the responses of the two groups to the individual statements was made through the Chi-Square test. In order to determine the numerical relationship in the Chi-square test, Phi values ranging between 0 and 1—indicating that the relationship increases as the relationship increases and decreases as the coincidence decreases—were also calculated. Finally, a backward multiple linear regression was used to identify possible predictors of the outcome overall stigma score out of the following candidate variables: Beck depression and anxiety scales, age, class of the participants, gender, having a mental health condition, using medicines related to any mental health condition, family having a mental health condition, and having a relationship with someone that used any psychotropic. At each step, variables were added based on *p*-values, and a *p*-value threshold of 0.2 was used to set a limit on the total number of variables included in the final model. The statistics were judged as significant at *p* < 0.05. IBM SPSS Statistics V 23 was used to conduct all statistical analyses.

## 3. Results

### 3.1. Demographics

In this study, 205 [first year: 104 (83.2%), fifth year: 101 (57.1%)] of a total of 302 (first year: 125, fifth year: 177) pharmacy students voluntarily completed the questionnaire between December 2022 and March 2023. All responses were included in the analyses. 

The majority of the participants (74.1%) were female. When the demographic data of the participants were compared, significant differences were detected between the first- and fifth-year student groups. Besides age, parents’ education level, smoking, having a mental health condition, using psychotropic drugs, and having a relationship with someone that used a psychotropic (*p* < 0.05) were different (Table 1). 

In terms of what was self-reported, 11.5% of the first-year students and 21.8% of the fifth-year students had any mental health condition, and 14.4% of the first-year students and 27.8% of the fifth-year students used any psychotropic. Although there was no significant difference between the two classes, the rate of having a session with a psychiatrist and psychologist was 24.0% vs. 33.7% and 31.7% vs. 40.6% for the first- and fifth-class students, respectively.

### 3.2. Scores on Depression, Anxiety, and DAKAS

Comparing the BDI and BAS scores of the students, the mean of both scores was higher for the fifth year students than for first year students, without reaching a statistically significant difference (*p* > 0.05). Related to the DAKAS questionnaire, the rates of accepting depression as a disease and antidepressant use as ‘common’ were 73.1% and 96.0% in the first- and fifth-year students, respectively. When the statements based on knowledge about the effects, adverse effects, mechanism, and use of antidepressants are analyzed, fifth- year students had a higher level of knowledge. The rate of agreement with the statement that antidepressant continuity is harmed by negative effects (stigmas) arising from negative perceptions of the society was 49.0% and 77.2% for the first- and fifth- year students, respectively. When considering the overall stigma scores, this was 21.61 and 15.89 for the first- and fifth-year students, respectively (*p* < 0.05) (Table 2 and Table 3). 

Starting with 9 variables that might theoretically be good predictors of the overall stigma score, a backward multiple logistic regression model was used to reduce them to 3, which were the following: being the fifth class and having a relationship with someone that used any psychotropic as reducing stigma, and male gender as increasing stigma. The overall stigma score of fifth-year students was 3.69 points lower than that of first-year students. This score in male students was 4.65 points higher than in female students. Also, the overall stigma score of students who have a relationship with someone that used a psychotropic was 3.06 points lower (Table 4).

## 4. Discussion 

Individuals with depression are in close interaction with healthcare professionals, including pharmacists. Patients may be exposed to direct stigmatization by healthcare professionals, or may experience internalized stigmatization due to the negative attitudes or substandard services and interactions of healthcare professionals. 

Studies have shown that the attitudes of healthcare professionals towards individuals with a mental health condition can indeed be negative [33,34,35,36]. Davis et al. asserted that the most effective type of intervention to decrease mental health stigma in students is social contact (working with patients who have mental health conditions) [37]. A study conducted in Japan found that psychiatric nurses had a negative opinion about the life of patients and their families in the ward [33,34]. In a study that included clinicians, patients, and patients’ relatives, it was observed that patients had the most negative beliefs about people with mental disease and clinicians were the ones who showed more fear and stigmatization behavior [35]. In another study, it was observed that the exclusionary and negative attitudes of health care professionals towards mental health conditions did not change in the last 10 years [36]. In studies that focused on the attitudes of students who intend to become health care workers towards mental health conditions, it was seen that students accept depression as a disease and have a positive perspective towards the phenomenon of depression, such as the study conducted on pharmacy students in Saudi Arabia [38]. However, in other studies, students reported negative attitudes [39,40]. 

It is known that 75% of common mental health conditions occur before the age of 25 [41]. In parallel with this information, in a study conducted among college students, it was found that the prevalence of depression was at a considerable level with a rate of 20% as mild, 16% as moderate, and 5% as severe [42]. In our study, according to the mean BDI and BAS scores, it was observed that the participants (<25 years of age) were classified as having a mild to moderate severity of anxiety and depression, in line with their subjective assessment.

Besides formal training in knowledge and skills, we claim that also informal peer communication and interaction over the 5 years of training is relevant. We suggest that informal interactions are likely instrumental to better understand the relevance of changing attitudes before starting the profession, and to consider the effect of negative attitudes. These negative attitudes relate to both the close contact of students with each other during the university period, and the close contact of these students with patients in society when they graduate. These changes obviously coincide while the students also evolve during their young adulthood. In this context, there are many studies examining the effect of education on stigmatization [43]. These studies generally focused on nursing and medical education, while we add similar observations in pharmacy students [44,45]. 

Having a mental health condition, using psychotropic drugs, and having a relationship with someone that used any psychotropic were more common in 5th year students than in 1st years, which was expected due to the age difference and the challenges of pharmacy education over time [14]. The fact that almost one in three students has consulted a psychiatrist or psychologist at least once shows that they care about their mental health and that, as a society, we should not neglect the mental health of future pharmacists.

Nalcakan et al. found that sixth (final)-year medical students were less concerned about the presence of conditions related to mental health conditions in their environment compared to first-year students (10.2% vs. 2%), the overall stigma score of sixth-year students was lower than first-year students (mean score = 23.0 vs. 16.0), and antidepressant use and depression awareness were higher in sixth-year medical students (68% vs. 98%) [10]. Similar results were obtained in our study. While 9.6% of the first-year pharmacy students were worried about someone around them using antidepressants, this rate was 2.0% for the fifth-year students. Also, there were significant differences between these student groups due to the accumulation of knowledge and skills. For example, while 54.8% of the first-year students accepted the statement that antidepressants are drugs that should be continued regularly, 85.1% of the fifth-year students accepted it. While the overall stigma score of the first-year students was 21.61, this score of the fifth-year students was 15.89 (*p* < 0.05). This suggests that education is impactful, and a driver of knowledge and skills. 

A survey study in which medical (18%), pharmacy (55%), and nursing (72%) students thought that antidepressants were useful for schizophrenia also revealed the importance of education on psychotropics in each discipline [46]. Intriguingly, pharmacists showed a lower level of knowledge about antidepressants compared to final-year pharmacy students [47]. In another study, it was observed that 10-question test scores related to mental health increased and stigma scores decreased in third-year pharmacy students who were trained against stigmatization related to mental health conditions after the training [48]. In contrast, there are also studies that reported on the absence of an impact of education and attitudes [49]. A questionnaire survey in medical students reported that there was no significant difference between second- and sixth-year students in their attitudes towards mental disease and that these attitudes were negative [50]. 

Based on these papers, we claim that providing fifth-year students with training on the use of psychotropic drugs and approaching these patients from a pharmacist’s perspective with various cases within the scope of the “Pharmaceutical Care” course during two weeks contributed positively to their attitudes and behaviors. It is a separate course taught by the Department of Clinical Pharmacy within the Faculty of Pharmacy. The course is based on 5 h of lectures and 3 h of case discussions (compulsory internship) per week during the autumn semester. In this course, a faculty member who has been actively participating in rounds and training as a psychiatric clinical pharmacist for many years supports the theoretical knowledge with examples encountered in the clinic. Also, fifth-year students examine and interpret clinical cases within the hospital as part of their internship program.

The limitation of the study is the low number of participants coming to pharmacy education from many regions of Turkey. The reason why the participation rate of the fifth class is lower than that of the first class is that the survey administered to them coincided with the Turkey–Syria earthquake, which deeply affected many regions of our country. Since all fifth-class participants completed the survey after the earthquake and we do not know whether they and/or their families were in the earthquake zone at the time of the earthquake, the effect of the earthquake on the questionnaire responses could not be analyzed, but is reasonable to be assumed. Since the survey was administered to the first class before the earthquake, these participants and their numbers were not affected by the earthquake. It is obviously also possible that the mind becomes more mature, with age serving as an influencing factor on the increased pharmaceutical knowledge. One limitation of the study was the inability to exclude the interference of these influencing factors. This causality assessment can be performed better in a prospective, longitudinal study, planned as a subsequent step of the current findings. 

## 5. Conclusions

Lower levels of stigma may have resulted from increased levels of pharmacy education, improved knowledge of mental health issues, and improved patient knowledge. One of the most essential methods to reduce stigma is to increase familiarity. There is an obvious influence of pharmacy education on a higher awareness and knowledge level and a lower stigma. While some facts should be highlighted more, the pharmacy curriculum teaches students to view mental health problems as diseases, their sufferers as patients, and antidepressants as a therapeutic option. Furthermore, because these ideas can be acquired through teaching, simulated education programs, public awareness campaigns/days, and clinical pharmacy education may aid in reducing stigmatizing attitudes.

## Figures and Tables

**Table 1:** The sociodemographic characteristics and responses on self-reported mental health conditions in the responding pharmacy students in relation to the year of study.

Characteristics	First Year (n = 104)	Fifth Year (n = 101)	*p*-Value
Age, mean (SD), years *	19.68 (0.87)	23.76 (1.64)	<0.001
Gender, n (%)			
*Female*	75 (72.1)	77 (76.2)	0.500
*Male*	29 (27.9)	24 (23.8)	
Number of siblings, mean (SD)	1.42 (1.00)	1.71 (1.23)	0.075
Family types *, n (%)			
*Nuclear*	99 (95.2)	84 (83.2)	
*Extended*	3 (2.9)	12 (11.9)	0.016
*Divorced*	2 (1.9)	5 (5.0)	
Father’s educational level, n (%)			
*Lower*	1 (1.0)	1 (1.0)	
*Middle*	42 (40.4)	49 (48.5)	0.163
*Higher*	61 (58.7)	51 (50.5)	
Mother’s educational level, n (%)			
*Lower*	3 (2.9)	3 (3.0)	
*Middle*	52 (49.9)	57 (56.4)	0.492
*Higher*	49 (47.1)	41 (40.6)	
Smoking *, n (%)			
*Never*	85 (81.7)	62 (61.4)	
*Quit*	9 (8.7)	16 (15.8)	0.005
*Active*	10 (9.6)	23 (22.8)	
Having a mental health condition *, n (%)	12 (11.5)	22 (21.8)	0.049
Using medicines related to a mental health condition *, n (%)	15 (14.4)	29 (28.7)	0.013
Having a relationship with someone that used any psychotropic *, n (%)	57 (54.8)	69 (68.3)	0.047
Having a family member with any mental health condition, n (%)	27 (26.0)	29 (28.7)	0.658
Beck depression scale, mean (SD)	14.16 (8.46)	16.55 (10.39)	0.166
*Min-Max score*	0–38	0–52	
Beck anxiety scale, mean (SD)	15.33 (11.57)	15.40 (11.06)	0.853
*Min-Max score*	0–46	0–52	
Having a session with a psychiatrist, n (%)	25 (24.0)	34 (33.7)	0.128
Having a session with a psychologist, n (%)	33 (31.7)	41 (40.6)	0.187

* *p* < 0.05 (statistically significant difference was found between the two groups), SD = standard deviation, n = number.

**Table 2:** Responses to the individual statements in Section A of the Depression and Antidepressant Knowledge and Awareness Scale (DAKAS) [10].

Section A	Strongly Agree + Agree, n (%)	Neutral, n (%)	Strongly Disagree + Disagree, n (%)
1-Depression is a disease and antidepressant use is common (*p* < 0.001 Phi = 0.322)			
1st year	76 (73.1)	17 (16.3)	11 (10.6)
5th year	97 (96.0)	1 (1.0)	3 (3.0)
2-Happinesses through antidepressant usage are fake happinesses (*p* < 0.001 Phi = 0.402)			
1st year	61 (58.7)	30 (28.8)	13 (12.5)
5th year	24 (23.8)	33 (32.7)	44 (43.6)
3-I think that people who use antidepressants are not strong enough (*p* = 0.264 Phi = 0.114)			
1st year	12 (11.5)	14 (13.5)	78 (75.0)
5th year	7 (6.9)	9 (8.9)	85 (84.2)
4-When/If I used antidepressants, I shared/would share it with my family (*p* = 0.847 Phi = 0.04)			
1st year	84 (80.8)	12 (11.5)	8 (7.7)
5th year	79 (78.2)	12 (11.9)	10 (9.9)
5-When/If I used antidepressants, I shared/would share it with my friends (*p* = 0.786 Phi = 0.048)			
1st year	61 (58.7)	30 (28.8)	13 (12.5)
5th year	64 (63.4)	26 (25.7)	11 (10.9)
6-Public stigma has a harmful effect on antidepressant drug continuance (*p* < 0.001 Phi = 0.331)			
1st year	51 (49.0)	57 (45.2)	6 (5.8)
5th year	78 (77.2)	15 (14.9)	8 (7.9)
7-Antidepressants are addictive (*p* < 0.001 Phi = 0.555)			
1st year	59 (56.7)	36 (34.6)	9 (8.7)
5th year	19 (18.8)	21 (20.8)	61 (60.4)
8-Antidepressants change the personality of the user (*p* = 0.004 Phi = 0.232)			
1st year	39 (37.5)	32 (30.8)	33 (31.7)
5th year	21 (20.8)	26 (25.7)	54 (53.5)
9-Psychological problems can be solved by willpower (*p* = 0.003 Phi = 0.237)			
1st year	57 (54.8)	30 (28.8)	17 (16.3)
5th year	35 (34.7)	31 (30.7)	35 (34.7)
10-Knowing somebody I know is using antidepressants would make me nervous (*p* = 0.037 Phi = 0.18)			
1st year	10 (9.6)	7 (6.7)	87 (83.7)
5th year	2 (2.0)	12 (11.9)	87 (86.1)
11-Knowing somebody I know is seeing a psychiatrist would make me nervous (*p* = 0.192 Phi = 0.135)			
1st year	6 (5.8)	4 (3.8)	94 (90.4)
5th year	1 (1.0)	3 (3.0)	97 (96.0)
12-Depression can only be treated by psychotherapy (*p* = 0.016 Phi = 0.201)			
1st year	29 (27.9)	43 (41.3)	32 (30.8)
5th year	13 (12.9)	43 (42.6)	45 (44.6)
13-I would be anxious to share that I am seeing a psychiatrist with my family (*p*= 0.134 Phi = 0.140)			
1st year	16 (15.4)	7 (6.7)	81 (77.9)
5th year	11 (10.9)	15 (14.9)	75 (74.3)
14-I would be anxious to share that I am seeing a psychiatrist with my friends (*p* = 0.036 Phi = 0.18)			
1st year	19 (18.3)	18 (17.3)	67 (64.4)
5th year	7 (6.9)	25 (24.8)	69 (68.3)
15-Antidepressants make people numb (*p* < 0.001 Phi = 0.326)			
1st year	52 (50.0)	35 (33.7)	17 (16.3)
5th year	31 (30.7)	23 (22.8)	47 (46.5)
16-I think people usually unnecessarily use the phrase ‘I am depressed.’ (*p* = 0.028 Phi = 0.923)			
1st year	91 (87.5)	6 (5.8)	7 (6.7)
5th year	88 (87.1)	7 (6.9)	6 (5.9)
17-I think the increased prevalence of depression is caused by increased awareness of depression (*p* = 0.462 Phi = 0.087)			
1st year	68 (65.4)	17 (16.3)	19 (18.3)
5th year	74 (73.3)	12 (11.9)	15 (14.9)
**Overall stigma score (formed by questions 1–14). *p *< 0.001, U = 2674.50, 1st year = 21.61, 5th year = 15.89**			

**Table 3:** Responses to the individual statements in Section B of the Depression and Antidepressant Knowledge and Awareness Scale (DAKAS) [10].

Section B	Strongly Agree + Agree, n (%)	Neutral, n (%)	Strongly Disagree + Disagree, n (%)
1-Depression has genetic elements (*p* = 0.064 Phi = 0.164)			
1st year	63 (60.6)	30 (28.8)	11 (10.6)
5th year	75 (74.3)	22 (21.8)	4 (4.0)
2-Antidepressants lower the sexual drive (*p* < 0.001 Phi = 0.387)			
1st year	22 (21.2)	80 (76.9)	2 (1.9)
5th year	49 (48.5)	40 (39.6)	12 (11.9)
3-Antidepressant usage is more common in females (*p* < 0.001 Phi = 0.325)			
1st year	38 (36.5)	52 (50.09	14 (13.5)
5th year	69 (68.3)	28 (27.7)	4 (4.0)
4-Antidepressants prevent the formation of clear thoughts (*p* < 0.001 Phi = 0.415)			
1st year	46 (44.2)	46 (44.2)	12 (11.5)
5th year	23 (22.8)	28 (27.7)	50 (49.5)
5-Antidepressants are only used for the treatment of depression (*p* < 0.001 Phi = 0.374)			
1st year	3 (2.9)	35 (33.7)	66 (63.5)
5th year	-	6 (5.9)	95 (94.1)
6-Antidepressants have negative effects on creativity (*p* < 0.001 Phi = 0.379)			
1st year	26 (25.0)	46 (44.2)	32 (30.8)
5th year	9 (8.9)	23 (22.8)	69 (68.3)
7-Antidepressants are drugs that require continuous use (*p* < 0.001 Phi= 0.344)			
1st year	57 (54.8)	27 (26.0)	20 (19.2)
5th year	86 (85.1)	5 (5.0)	10 (9.9)
8-Antidepressants show their effects after a short period (*p* < 0.001 Phi = 0.516)			
1st year	18 (17.3)	43 (41.3)	43 (41.3)
5th year	1 (1.0)	9 (8.9)	91 (90.1)
9-All antidepressants aim to increase levels of serotonin (*p* < 0.001 Phi = 0.471)			
1st year	9 (8.7)	54 (51.9)	41 (39.4)
5th year	11 (10.9)	9 (8.9)	81 (80.2)
10-Antidepressants cannot be purchased without a prescription (*p* < 0.001 Phi = 0.414)			
1st year	75 (72.1)	21 (20.2)	8 (7.7)
5th year	53 (52.5)	6 (5.9)	42 (41.6)
11-There is no need to consult a doctor for the use of plant-based sedatives (*p* = 0.087 Phi = 0.154)			
1st year	2 (1.9)	9 (8.7)	93 (89.4)
5th year	1 (1.0)	2 (2.0)	98 (97.0)
12-Antidepressants cannot be used in ages 0–6 (*p* < 0.001 Phi = 0.29)			
1st year	54 (51.9)	40 (38.5)	10 (9.6)
5th year	32 (31.7)	37 (36.6)	32 (31.7)

**Table 4:** Regression analysis on the predictors of the overall stigma score [10].

Predictors	B (Odds Ratio)	SE	Beta	t	*p*
Beck depression scale	−0.05	0.06	−0.07	−0.08	0.406
Beck anxiety scale	0.08	0.05	0.13	1.60	0.110
Age	−0.34	0.33	−0.11	−1.03	0.302
Class of the participants (5th year)	−3.69	1.63	−0.25	−2.26	0.025 *
Gender (male)	4.65	1.05	0.28	4.42	<0.001 *
Having a mental health condition	−2.37	1.72	−0.12	−1.38	0.169
Using medicines related to any mental health condition	1.52	1.55	0.08	0.98	0.328
Family having a mental health condition	−0.48	1.24	−0.03	−0.39	0.697
Having a relationship with someone that used any psychotropic	−3.06	1.13	−0.21	−2.68	0.008 *

* *p* < 0.05, SE: Standard error.

## Data Availability

The anonymous data presented in this study are available upon reasonable request from the corresponding author. The data are not publicly available due to privacy and ethical restrictions.

## Supplementary Materials

The following supporting information can be downloaded at: https://www.mdpi.com/article/10.3390/pharmacy12020045/s1, Table S1: Checklist for Reporting Results of Internet E-Surveys (CHERRIES).
